# Perception of Smile Attractiveness Based on Upper Dental Midline Shift and Gingival Display Among Dental Professionals and General Population: A Questionnaire-Based Study

**DOI:** 10.7759/cureus.99173

**Published:** 2025-12-13

**Authors:** Harshini P. R., Rajesh R. N. G., Rony Kondody, Anushree M. K., Roopa K.

**Affiliations:** 1 Orthodontics and Dentofacial Orthopedics, Sri Rajiv Gandhi College of Dental Sciences and Hospital, Bengaluru, IND; 2 Orthodontics and Dentofacial Orthopaedics, Sri Rajiv Gandhi College of Dental Sciences and Hospital, Bengaluru, IND

**Keywords:** gingival display, micro aesthetics, smile attractiveness, smile esthetics, upper dental midline shift

## Abstract

Aim

To assess and compare the perception of smile attractiveness based on upper dental midline shifts and gingival display among dental professionals and the general population.

Methodology

A cross-sectional questionnaire-based study was conducted after obtaining ethical approval (SRGCDS/2025/105). A total of 106 participants aged 20-50 years were recruited, comprising 27 dental professionals and 79 members of the general population. A standardized high-resolution smile photograph was digitally altered to simulate varying upper dental midline shifts (0, 1.5, and 3 mm) and gingival display levels (0, 2, and 4 mm). Participants evaluated the attractiveness of the images through a structured online questionnaire, selecting their responses from predefined options for each image. Data were analyzed using SPSS version 20.0 with descriptive statistics, chi-square tests, analysis of variance (ANOVA), and Cohen’s kappa for reliability. A *P*-value < 0.05 was considered statistically significant.

Results

Smiles with minimal midline deviation and 0-2 mm gingival display were rated most attractive. Dental professionals were significantly more critical of midline shifts and excessive gingival display compared to the general population (*P *= 0.04). They more frequently identified 3 mm deviations as unattractive, requiring correction, and associated them with long-term impacts (*P *= 0.03). Both groups rated the 4 mm gingival display least attractive. Over half of the participants (60, 56.6%) prioritized correcting both midline and gingival discrepancies simultaneously.

Conclusions

Perceptions of smile attractiveness are influenced by both upper dental midline alignment and gingival display. Dental professionals demonstrate greater sensitivity to deviations than the general population. Recognizing these perceptual differences is essential for clinicians to align treatment goals with patient expectations, thereby enhancing aesthetic and functional outcomes in orthodontic treatment.

## Introduction

Smile aesthetics plays an assertive role in the success of orthodontic treatment, influencing not only facial harmony but also social interactions and self-esteem. A well-balanced and attractive smile is a key component of overall facial aesthetics, impacting first impressions and social confidence [1]. Among the various factors that contribute to smile perception, two crucial elements are the alignment of the upper dental midline and the visibility of gingival tissue during a smile.

The upper dental midline refers to the vertical alignment of the maxillary central incisors relative to the facial midline. Ideally, this alignment should coincide with the midline of the face; however, deviations are commonly observed in clinical practice. Even minor discrepancies in the upper dental midline can influence smile symmetry and perceived attractiveness [2]. Research suggests that midline shifts greater than 2 mm become noticeable to both dental professionals and laypersons, though professionals are often more critical in their assessments [3]. A recent study published in 2024 found that dental professionals detected a significant loss of attractiveness with a 1 mm upper midline shift in female subjects, while laypersons noticed this change at 2 mm. Notably, both groups were more sensitive to midline deviations in female models, with dental professionals generally more discerning in their evaluations [4].

Another significant factor in smile aesthetics is gingival display, which refers to the amount of gum tissue visible when smiling. An ideal smile presents a harmonious balance between teeth, lips, and gingiva. Excessive gingival display, often termed a "gummy smile," or minimal gingival display can affect the perceived appeal of a smile [5]. While dental professionals rely on precise measurements and clinical parameters, laypersons typically base their judgments on overall facial aesthetics rather than specific dental details [6]. A 2023 study assessing the influence of gingival exposure on smile attractiveness among Saudi Arabian dental professionals and laypersons revealed that both groups rated smiles with a 1-2 mm gingival display as the most attractive, whereas a 4 mm gingival display was deemed the least appealing [7].

Perceptions of smile aesthetics vary between dental professionals and the general population due to differences in education, experience, and awareness of ideal occlusion and facial symmetry [8]. However, most of the existing literature has been conducted among Western and Middle Eastern populations, with limited data available among Indian subjects. Since cultural, ethnic, and regional factors can significantly influence perceptions of facial and dental aesthetics, findings from other populations may not be directly applicable to the Indian context.

Therefore, this study aims to evaluate the influence of predetermined amounts of upper dental midline shift (0, 1.5, and 3 mm) and gingival display (0, 2, and 4 mm) on perceived smile attractiveness. The perceptions of dental professionals and laypersons will be compared using standardized, digitally altered smile photographs. By analyzing these variables within an Indian population, the study seeks to provide culturally relevant data that can contribute to establishing aesthetic reference standards and support clinical decision-making in orthodontic diagnosis and treatment planning.

## Materials and methods

This study followed a cross-sectional design aimed at assessing how variations in the upper dental midline and gingival display influence the perception of smile attractiveness. It was carried out with prior approval from the Ethical Committee, Sri Rajiv Gandhi College of Dental Sciences and Hospital [SRGCDS/2025/105].

The sample size was calculated using G*Power software (version 3.1.9.7), drawing on findings from Pinho et al. [2], who reported a moderate effect size when evaluating esthetic impacts of midline deviations. Based on an effect size of 0.25, an alpha value of 0.05, and a statistical power of 80%, the minimum required sample was 106 participants. The effect size used for this calculation was derived from the partial eta-squared value ($\eta_p^2 \approx 0.06$) reported by Pinho et al., which was converted to Cohen’s f using the formula:

\[ f = \sqrt{\frac{\eta_p^2}{1 - \eta_p^2}} \].

Substituting $\eta_p^2 \approx 0.06$ yields *f* ≈ 0.25, which was used as the effect size parameter for the present study. The final total sample size included in the study was 106 participants.

Participants were grouped as either dental professionals (including dentists, orthodontists, and other specialists with formal dental training) or members of the general population (individuals without any dental background, from varied age groups and professions). Inclusion criteria included an age range of 20-50 years, absence of visual impairments, and informed consent. To ensure consistency during data collection, the principal investigator (PI) underwent specific training in smile aesthetics evaluation, supervised by a senior orthodontist. Calibration was performed using a sample of ten participants, and the PI’s assessments were compared with those of two experienced clinicians. Inter-examiner reliability was calculated using Cohen’s kappa (κ), with values above 0.80 indicating strong agreement.

One standardized high-resolution smile photograph served as the base image for digital modification. Adobe Photoshop CC 2024 was used to create variations by shifting the upper dental midline (0, 1.5, and 3 mm) and altering the gingival display (0, 2, and 4 mm). The lighting, background, and image quality were kept uniform across all versions. The manipulated images were exported in JPEG format at full resolution and embedded in a structured Google Forms questionnaire. The questionnaire was specifically developed for this study and underwent face and content validation by three dental professionals (two orthodontists and one prosthodontist), who evaluated the clarity, relevance, and appropriateness of both the items and the digital stimuli (Appendix). A pilot test involving 15 participants (not included in the final sample) was conducted to assess comprehension and response consistency, after which minor wording adjustments were made. Internal consistency was measured using Cronbach’s alpha, with values above 0.7 indicating acceptable reliability.

**Figure 1:**
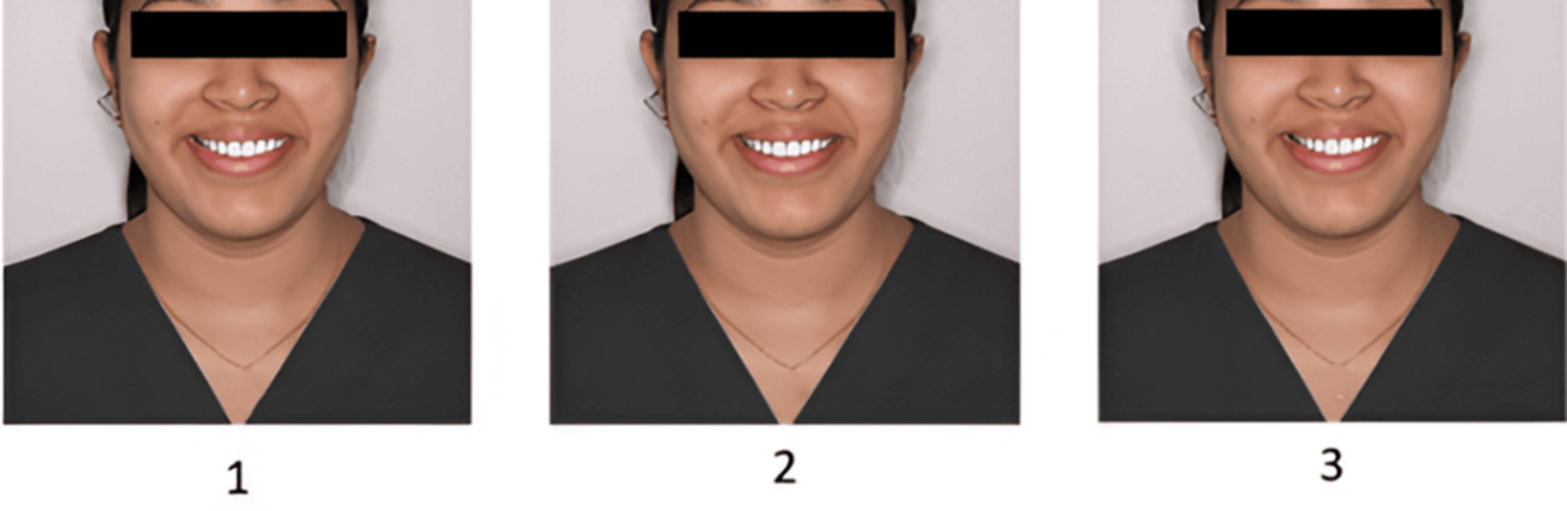
Sequence 1: Image 1 - gingival display 0 mm, midline shift 0 mm; Image 2 - gingival display 0 mm, midline shift 1.5 mm; Image 3 - gingival display 0 mm, midline shift 3 mm.

**Figure 2:**
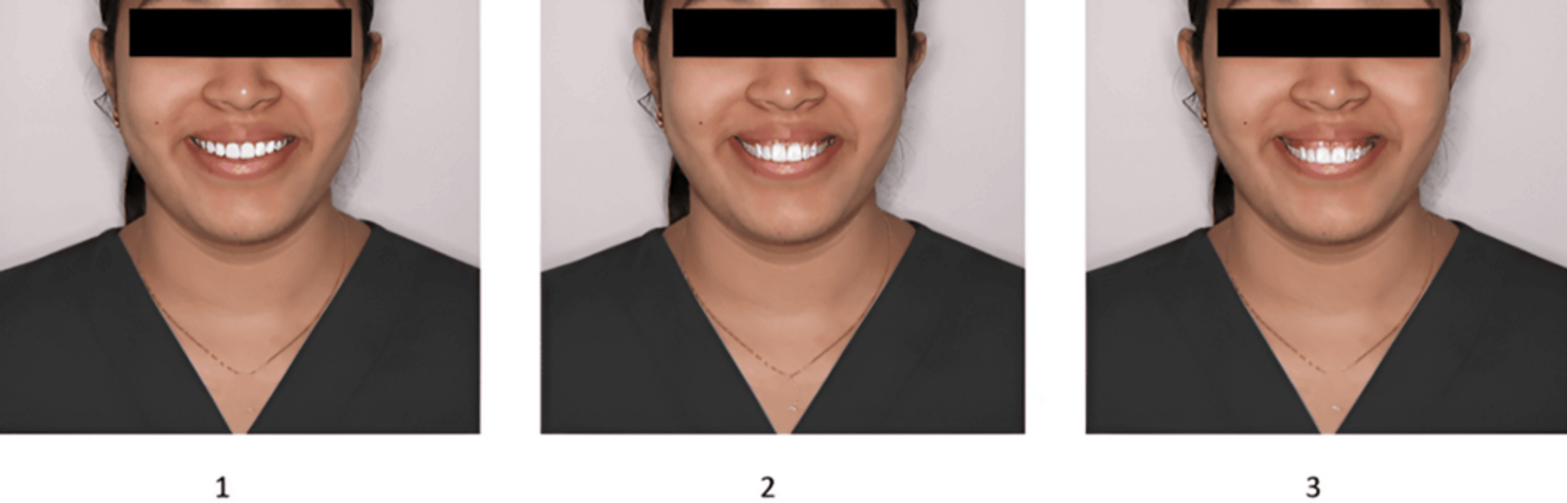
Sequence 2: Image 1 - gingival display 0 mm, midline shift 0 mm; Image 2 - gingival display 2 mm, midline shift 0 mm; Image 3 - gingival display 4 mm, midline shift 0 mm.

**Figure 3:**
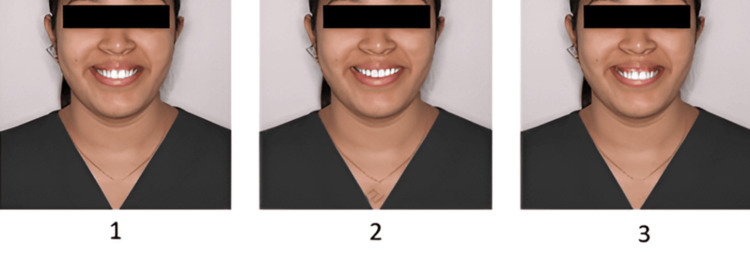
Sequence 3: Image 1 - gingival display 2 mm, midline shift 1.5 mm; Image 2 - gingival display 0 mm, midline shift 1.5 mm; Image 3 - gingival display 4 mm, midline shift 1.5 mm.

**Figure 4:**
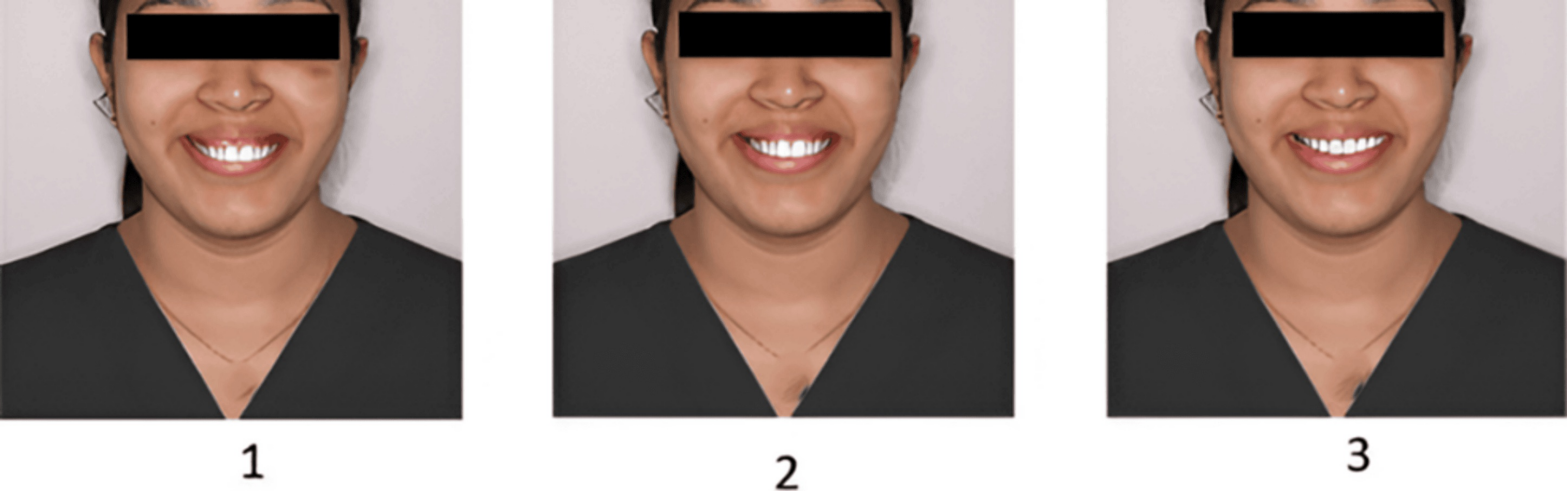
Sequence 4: Image 1 - gingival display 4 mm, midline shift 3 mm; Image 2 - gingival display 2 mm, midline shift 3 mm; Image 3 - gingival display 0 mm, midline shift 3 mm.

The finalized questionnaire contained four sets of images, with three photos per set (Figures 1-4). Sequence 1 included smiles with increasing midline deviation and no gingival display alteration. Sequence 2 presented a varying gingival display without midline shift. Sequence 3 combined a 1.5 mm midline shift with different gingival display levels, and Sequence 4 combined a 3 mm midline shift with varying gingival displays. Each set was followed by six questions designed to assess how the smile was perceived in terms of attractiveness, whether midline shifts or gingival visibility were noticed, and preferences regarding ideal smile features. The survey was distributed online via Google Forms using a random circulation method through social media and messaging platforms. All participants provided informed consent before taking part in the study. Participation was voluntary, with no incentives offered, and responses were anonymized to maintain confidentiality.

Data were analyzed using SPSS software (version 20.0; IBM Corp., Armonk, NY). Descriptive statistics summarized participant demographics and responses. Cronbach’s alpha was used to assess internal consistency, with values above 0.7 considered acceptable. One-way ANOVA was used to compare ratings across different midline and gingival display levels, while chi-square tests evaluated categorical data. Cohen’s kappa was used for reliability testing, with κ > 0.80 indicating excellent agreement. Before performing inferential analysis, the distribution of continuous variables was examined using the Shapiro-Wilk test. The data met assumptions of normality (*P* > 0.05), supporting the use of parametric tests. A *P*-value of less than 0.05 was considered statistically significant.

## Results

A total of 106 participants were included in the study. Of these, 52 were males (49.1%) and 54 were females (50.9%). The largest age group was 20-30 years (64, 60.4%), followed by 30-40 years (29, 27.4%) and 40-50 years (13, 12.3%). Dental professionals comprised 27 (25.5%), while the general population represented 79 (74.5%). This distribution ensured adequate diversity in both professional background and age (Table 1).

**Table 1:** Sociodemographic characteristics of participants. Data presented as absolute numbers (*n*) and percentages (%).

Characteristic	Category	n	%
Age group (years)	20-30	64	60.4
	30-40	29	27.4
	40-50	13	12.3
Gender	Male	52	49.1
	Female	54	50.9
Occupation	Dental professionals	27	25.5
	General population	79	74.5

When evaluating smiles with midline deviations, participants showed clear preferences. Half of them (53, 50%) selected Image 2 as the most aesthetically pleasing, while Image 3 was rated least attractive by ( 47, 44.3%). Image 3 was also most frequently perceived as showing the greatest deviation (42, 39.6%) and as making the teeth appear unbalanced (46, 43.4%). A total of 40 (37.7%) participants indicated that Image 3 required correction. Regarding long-term impacts, 37 (34.9%) believed that midline deviations could affect oral health, while others were unsure. Statistically significant differences were observed between groups: dental professionals were more likely than laypersons to identify Image 3 as least aesthetic (*P* = 0.04), to state that it required correction (*P* = 0.04), and to report that it could affect long-term dental health (*P* = 0.03) (Table 2).

**Table 2:** Comparison of responses to upper dental midline shift on smile attractiveness in Image Sequence 1 based on participants’ occupation (chi-square test). ^*^P-value <0.05 - statistically significant.

Question	Response/Image	Dental professionals, *n* (%)	Others, *n* (%)	*χ*²-value	*P*-value
Which image has the most aesthetically pleasing smile?	Image 1	8 (29.6%)	24 (30.4%)	2.01	0.37
	Image 2	16 (59.3%)	37 (46.8%)		
	Image 3	3 (11.1%)	18 (22.8%)		
Which image has the least aesthetically pleasing smile?	Image 1	4 (14.8%)	30 (38.0%)	6.23	0.04*
	Image 2	6 (22.2%)	19 (24.1%)		
	Image 3	17 (63.0%)	30 (38.0%)		
Which has the most noticeable midline deviation?	Image 1	9 (33.3%)	20 (25.3%)	0.66	0.72
	Image 2	8 (29.6%)	27 (34.2%)		
	Image 3	10 (37.0%)	32 (40.5%)		
Midline shift causing teeth to look unbalanced?	Image 1	6 (22.2%)	27 (34.2%)	1.36	0.51
	Image 2	8 (29.6%)	19 (24.1%)		
	Image 3	13 (48.1%)	33 (41.8%)		
Most requiring correction for midline shift?	Image 1	11 (40.7%)	21 (26.6%)	1.95	0.04*
	Image 2	7 (25.9%)	27 (34.2%)		
	Image 3	9 (33.3%)	31 (39.2%)		
Perception of long-term impacts of midline shifts on dental health?	Yes	15 (55.6%)	22 (27.8%)	6.84	0.03*
	No	4 (14.8%)	17 (21.5%)		
	Maybe	8 (29.6%)	40 (50.6%)		

For Sequence 2, in which the gingival display was altered, Image 1 was selected as the most aesthetic by 82 (79.2%) participants, while Image 3 was rated as the least attractive by 75 (70.8%). Excessive gingival display in Image 3 was considered to have the most negative impact by 79 (74.5%) participants and was most frequently judged as requiring correction by 72 (67.9%). Image 1 was perceived as having the most balanced tooth-gingiva proportion by 73 (68.9%). Importantly, dental professionals were significantly more critical of gingival display than the general population (*P* = 0.04) (Table 3).

**Table 3:** Comparison of responses to gingival display on smile attractiveness in Image Sequence 2 based on participants’ occupation (chi-square test). *P-value < 0.05 - statistically significant.

Question	Response/Image	Dental professionals, *n* (%)	Others, *n* (%)	*P*-value	Chi-square value
Which image has the most aesthetically pleasing smile?	Image 1	22 (81.5%)	62 (78.5%)	0.22	5.7
	Image 2	1 (3.7%)	11 (13.9%)		
	Image 3	4 (14.8%)	6 (7.6%)		
Which image has the least aesthetically pleasing smile?	Image 1	2 (7.4%)	13 (16.5%)	0.16	6.52
	Image 2	2 (7.4%)	14 (17.7%)		
	Image 3	23 (85.2%)	52 (65.8%)		
Gum display has the greatest negative impact	Image 1	0 (0.0%)	14 (17.7%)	0.04*	7.2
	Image 2	4 (14.8%)	9 (11.4%)		
	Image 3	23 (85.2%)	56 (70.9%)		
Most balanced teeth-to-gum relationship	Image 1	18 (66.7%)	55 (69.6%)	0.92	0.18
	Image 2	5 (18.5%)	12 (15.2%)		
	Image 3	4 (14.8%)	12 (15.2%)		
Requires most reduction of gum display	Image 1	3 (11.1%)	11 (13.9%)	0.16	3.99
	Image 2	2 (7.4%)	18 (22.8%)		
	Image 3	22 (81.5%)	50 (63.3%)		
Which aspect would you prioritize for treatment?	Midline	1 (3.7%)	7 (8.9%)	0.26	2.96
	Gum display	9 (33.3%)	21 (26.6%)		
	Both	17 (63.0%)	43 (54.4%)		
	None of the above	0 (0.0%)	8 (10.1%)		

In Sequence 3, the majority of participants perceived Image 2 as the most aesthetically pleasing (84, 79.2%). Image 3, on the other hand, was rated as the least attractive (71, 67.0%) and was considered to have the greatest negative impact (72, 67.9%). It was also identified as most in need of correction (74, 69.8%). Image 2 was viewed as having the most balanced smile by 66 participants (62.3%). Statistically significant differences were observed between dental professionals and laypersons in their perception of balance (*P *= 0.04), with dental professionals showing more sensitivity to gingival proportions (Table 4).

**Table 4:** Comparison of responses to gingival display on smile attractiveness in Image Sequence 3 based on participants’ occupation using the chi-square test. *P-value < 0.05 - statistically significant.

Question	Response/Image	Dental professionals, *n* (%)	Others, *n* (%)	*P*-value	Chi-square value
Which image has the most aesthetically pleasing smile?	Image 1	4 (14.8%)	7 (8.9%)	0.32	2.79
	Image 2	22 (81.5%)	62 (78.5%)		
	Image 3	1 (3.7%)	10 (12.7%)		
Which image has the least aesthetically pleasing smile?	Image 1	3 (11.1%)	14 (17.7%)	0.63	1.17
	Image 2	4 (14.8%)	14 (17.7%)		
	Image 3	20 (74.1%)	51 (64.6%)		
In which image does gum display the greatest negative impact?	Image 1	3 (11.1%)	15 (19.0%)	0.44	2.06
	Image 2	3 (11.1%)	13 (16.5%)		
	Image 3	21 (77.8%)	51 (64.6%)		
Which smile has most balanced teeth-to-gum relationship?	Image 1	3 (11.1%)	20 (25.3%)	0.04*	7.82
	Image 2	22 (81.5%)	44 (55.7%)		
	Image 3	2 (7.4%)	15 (19.0%)		
Which image requires the most reduction of gum display?	Image 1	1 (3.7%)	11 (13.9%)	0.24	2.71
	Image 2	4 (14.8%)	16 (20.3%)		
	Image 3	22 (81.5%)	52 (65.8%)		
Which aspect would you prioritize for treatment?	Midline	1 (3.7%)	10 (12.7%)	0.11	3.67
	Gum Display	5 (18.5%)	19 (24.1%)		
	Both	21 (77.8%)	43 (54.4%)		
	None of the above	0 (0.0%)	7 (8.9%)		

In Sequence 4, Image 3 emerged as the most aesthetic option (79, 74.5%) and was considered to demonstrate the most balanced tooth-gingiva relationship (68, 64.2%). Conversely, Image 1 was rated as the least attractive (78, 73.6%) and as most in need of gingival reduction (69, 65.1%). The excessive gingival display in Image 1 was perceived as having the greatest negative impact (74, 69.8%). No statistically significant differences were observed between dental professionals and laypersons in this sequence (Table 5).

**Table 5:** Comparison of responses to gingival display on smile attractiveness in Image Sequence 4 based on participants’ occupation (chi-square test). *P-value < 0.05 - statistically significant.

Question	Response/Image	Dental professionals, *n* (%)	Others, *n* (%)	*P*-value	Chi-square value
Which image has the most aesthetically pleasing smile?	Image 1	1 (3.7%)	11 (13.9%)	0.27	3.84
	Image 2	3 (11.1%)	12 (15.2%)		
	Image 3	23 (85.2%)	56 (70.9%)		
Which image has the least aesthetically pleasing smile?	Image 1	22 (81.5%)	56 (70.9%)	0.24	4.74
	Image 2	2 (7.4%)	17 (21.5%)		
	Image 3	3 (11.1%)	6 (7.6%)		
In which image does gum display the greatest negative impact?	Image 1	23 (85.2%)	51 (64.6%)	0.13	5.46
	Image 2	2 (7.4%)	17 (21.5%)		
	Image 3	2 (7.4%)	11 (13.9%)		
Which smile has the most balanced teeth-to-gum relationship?	Image 1	5 (18.5%)	16 (20.3%)	0.92	0.34
	Image 2	5 (18.5%)	12 (15.2%)		
	Image 3	17 (63.0%)	51 (64.6%)		
Which image requires the most reduction of gum display?	Image 1	20 (74.1%)	49 (62.0%)	0.53	2.80
	Image 2	5 (18.5%)	21 (26.6%)		
	Image 3	2 (7.4%)	9 (11.4%)		
Which aspect would you prioritize for treatment?	Midline	3 (11.1%)	6 (7.6%)	0.13	4.81
	Gum Display	5 (18.5%)	25 (31.6%)		
	Both	19 (70.4%)	40 (50.6%)		
	None of the above	0 (0.0%)	8 (10.1%)		

A pooled comparison across all sequences revealed that dental professionals were consistently stricter in their aesthetic judgments. They were more likely to consider midline deviations as unaesthetic (*P *= 0.04), to recommend correction for midline shifts (*P* = 0.04), and to associate deviations with long-term impacts (*P *= 0.03). They also rated gingival display more critically, particularly in Sequence 2 (*P *= 0.04), and expressed different views on balanced smiles in Sequence 3 (*P *= 0.04). These findings highlight the professional sensitivity of dentists compared to the general population (Table 6).

**Table 6:** Statistically significant differences between dental professionals and the general population using the chi-square test. *P < 0.05 statistically significant. Percentages indicate relative proportions; wording reflects comparative trends rather than absolute values.

Variable	Dental professionals (%)	General population (%)	*χ*²-value	*P*-value
Least aesthetic smile (Midline deviation)	63 (Image 3)	38 (Images 1 and 3)	4.2	0.04*
Requires correction (Midline deviation)	40.7 (Image 1)	39.2 (Image 3)	4.2	0.04*
Long-term impact (Midline deviation)	55.6 (Yes)	50.6 (Maybe)	4.7	0.03*
Negative impact of gingival display (Sequence 2)	85.2 (Image 3)	70.9 (Image 3)	4.2	0.04*
Balanced smile (Sequence 3)	81.5 (Image 2)	55.7 (Image 2)	4.2	0.04*

When asked about treatment priorities, 60 (56.6%) participants felt that both midline alignment and gingival display should be corrected together. Gingival display alone was prioritized by 30 (28.3%) participants, while only 16 (15.1%) believed that midline correction alone should be addressed. Dental professionals showed a greater tendency to prioritize combined correction compared to the general population, suggesting their holistic approach to smile aesthetics (Table 7).

**Table 7:** Treatment priorities reported by participants (combined Sequences 2-4). More than half of the participants preferred simultaneous correction of both the midline and gingival display.

Treatment priority	n	%
Both midline and gingival display	60	56.6
Gingival display only	30	28.3
Midline only	16	15.1

## Discussion

The evaluation of smile aesthetics has an important role not only in dental appearance but also in social interactions, confidence, and overall facial attractiveness. Because of this, understanding how different people judge smile features has become increasingly important in modern dentistry. Smile analysis requires considering multiple factors, including midline position, gingival display, tooth shape, and lip dynamics, as described by Sarver and Ackerman [1]. Hence, the present study focuses on upper dental midline deviation and gingival display, two elements known to strongly influence how a smile is perceived.

Our findings showed that smiles with small midline deviations were rated as more attractive, while larger deviations were judged negatively. This agrees with Pinho et al. [2], who found that midline deviations greater than 2 mm were easily detected and considered unattractive. Similarly, Kokich et al. [3] demonstrated that even subtle dental asymmetries can affect smile attractiveness. Recent literature also supports that noticeable midline shifts negatively influence facial esthetics, including work by Sayahpour et al. [4], who reported reduced attractiveness with increasing dental midline shift. Ayyildiz et al. [5] also noted that dental students were sensitive to midline changes, reinforcing the importance of midline alignment in esthetic judgment. Additional research by Ioi et al. [6] showed that the vertical positioning of anterior teeth affects esthetic evaluation, which further supports the sensitivity of observers to changes in the dental midline region.

Gingival display also played a significant role in esthetic perception. In our study, 0 to 2 mm of gingival display was preferred, while 4 mm or more was perceived as unattractive. This is consistent with findings from Alaqeely et al. [7], who observed that both professionals and laypersons rated excessive gingival display as unesthetic. Rosa et al. [8] similarly reported that altered dental features, including gingival display, were judged more harshly by dental professionals. Johnston et al. [9] also found that deviations affecting the harmony between dental and facial midlines influenced attractiveness ratings. Ioi et al. [10] further showed that gingival display preferences differ across populations, highlighting cultural influences. Abu Alhaija et al. [11] supported these findings by demonstrating that orthodontists generally use stricter criteria than laypersons when evaluating smile esthetics.

Past research also confirms that laypersons tolerate small variations more readily than dental professionals. Ker et al. [12] reported that laypersons were less sensitive to minor esthetic changes compared to clinicians. Pithon et al. [13] similarly found that laypersons did not notice small midline deviations unless they were obvious. Geron and Atalia [14] further showed that increased gingival display negatively impacts attractiveness, especially when more than 3 or 4 mm of gingiva is visible. Pinzan-Vercelino et al. [15] also found that gingival display significantly influences attractiveness ratings, with 4 mm or more considered unattractive in most cases. The esthetic impact of smile components is supported by classic work from Dong et al. [16], who explained that a harmonious smile requires balanced dental and facial components.

Additional research helps explain individual variations in aesthetic judgment. Chaves et al. [17] observed that people differ in how much gingival display they find acceptable. Moore et al. [18] showed that buccal corridor width can also influence smile esthetics, demonstrating that many features interact to affect the overall perception of attractiveness. Parekh et al. [19] reported that variations in the smile arc and buccal corridor space influence how youthful and pleasing a smile appears. Finally, Pausch et al. [20] demonstrated that male and female observers may judge gingival exposure differently, which further supports the complex, multifactorial nature of smile perception.

Overall, the findings of the present study are consistent with this broader literature and show that both dental professionals and laypersons prefer smiles with minimal midline deviation and limited gingival display. However, professionals tend to be more critical and more sensitive to subtle changes than laypersons. This difference suggests that dental clinicians should consider both professional norms and patient preferences when planning esthetic treatment. Communication, visual simulations, and shared decision making can help ensure that treatment goals reflect what the patient considers important rather than focusing only on clinical perfection.

These results have practical clinical implications. Small midline deviations or mild gingival display may not require correction if the patient does not perceive them as unattractive. In contrast, larger deviations or excessive gingival exposure may justify orthodontic, periodontal, or restorative intervention. Understanding how patients perceive their own smile can help clinicians avoid unnecessary overtreatment, reduce patient anxiety, and achieve outcomes that improve satisfaction and confidence.

Future research should include multiple facial models with varied ethnic backgrounds, facial types, and smile characteristics to improve generalizability. Dynamic assessments, such as videos or three-dimensional imaging, would better represent natural smiling and allow more accurate esthetic evaluation. Additional variables such as tooth color, smile arc, and lip dynamics should also be explored to better understand how different esthetic components interact.

Although the study provides meaningful insights, certain limitations should be considered. The use of digitally modified two-dimensional images does not reflect the natural movement of a real smile. Only one facial model was drawn from a single region, and cultural differences, which are known to influence esthetic perception [7,10,15], may affect how applicable the results are to other populations. Finally, aesthetic evaluation is subjective, and individual preferences may affect ratings even when reliability is strong. These factors should be considered while designing future studies.

## Conclusions

Upper dental midline deviation and gingival display play a major role in perceived smile attractiveness. Smiles with minimal midline displacement (≤1.5 mm) and moderate gingival exposure (0-2 mm) were most attractive, and larger displacement or extreme gingival display (≥4 mm) was least attractive. Dentists were more attuned to small deviations than laypersons, who were more concerned with facial harmony, underlining the influence of clinical training on aesthetic perception.

These observations highlight the need for balancing aesthetic treatment and patient expectations in order to ensure satisfactory outcomes. Technical competence and interpersonal communication skills are both necessary for success in esthetic dentistry. Future studies using dynamic smile assessments, larger and more diverse populations, and cross-cultural comparisons are recommended to further guide evidence-based aesthetic treatment planning.

## Appendices

Appendix

**Table 8:** Treatment priorities reported by participants (combined Sequences 2-4). More than half of the participants preferred simultaneous correction of both the midline and gingival display.

Section	Question / Item	Response Options
Demographic Details	Age	20–30 | 30–40 | 40–50
	Gender	Male | Female | Prefer not to say
	Occupation	Dental Professional | Layperson / Others
Image Sequence 1 (Midline Deviation)	Most aesthetically pleasing smile	1 | 2 | 3
	Least aesthetically pleasing smile	1 | 2 | 3
	Most noticeable upper dental midline deviation	1 | 2 | 3
	Midline shift causing unbalanced appearance	1 | 2 | 3
	Photograph requiring definite correction	1 | 2 | 3
	Long-term impact on dental health/tooth wear	Yes | No | Maybe
Image Sequence 2 (Gingival Display)	Most aesthetically pleasing smile	1 | 2 | 3
	Least aesthetically pleasing smile	1 | 2 | 3
	Greatest negative impact of excessive gum display	1 | 2 | 3
	Most balanced teeth–gum relationship	1 | 2 | 3
	Greatest need for gingival reduction	1 | 2 | 3
	Treatment priority	Midline | Gum Display | Both | None
Image Sequence 3 (Combined Assessment)	Most aesthetically pleasing smile	1 | 2 | 3
	Least aesthetically pleasing smile	1 | 2 | 3
	Greatest negative impact of excessive gum display	1 | 2 | 3
	Most balanced teeth–gum relationship	1 | 2 | 3
	Greatest need for gingival reduction	1 | 2 | 3
	Treatment priority	Midline | Gum Display | Both | None
Image Sequence 4 (Combined Assessment)	Most aesthetically pleasing smile	1 | 2 | 3
	Least aesthetically pleasing smile	1 | 2 | 3
	Greatest negative impact of excessive gum display	1 | 2 | 3
	Most balanced teeth–gum relationship	1 | 2 | 3
	Greatest need for gingival reduction	1 | 2 | 3
	Treatment priority	Midline | Gum Display | Both | None
